# Levels of Key Enzymes of Methionine-Homocysteine Metabolism in Preeclampsia

**DOI:** 10.1155/2013/731962

**Published:** 2013-08-20

**Authors:** Alejandra Pérez-Sepúlveda, Pedro P. España-Perrot, Ximena Fernández B, Verónica Ahumada, Vicente Bustos, José Antonio Arraztoa, Aneta Dobierzewska, Horacio Figueroa-Diesel, Gregory E. Rice, Sebastián E. Illanes

**Affiliations:** ^1^Department of Obstetrics and Gynecology, Faculty of Medicine, Universidad de los Andes, San Carlos de Apoquindo, 7620001 Santiago, Chile; ^2^Clinical Perinatal Unit, Clínica Dávila, 8431657 Santiago, Chile; ^3^The University of Queensland, Centre for Clinical Research, RBWH Campus, Herston, Brisbane, QLD 4029, Australia

## Abstract

*Objective*. To evaluate the role of key enzymes in the methionine-homocysteine metabolism (MHM) in the physiopathology of preeclampsia (PE). *Methods*. Plasma and placenta from pregnant women (32 controls and 16 PE patients) were analyzed after informed consent. Protein was quantified by western blot. RNA was obtained with RNA purification kit and was quantified by reverse transcritase followed by real-time PCR (RT-qPCR). Identification of the C677T and A1298C methylenetetrahydrofolate reductase (MTHFR) single-nucleotide polymorphisms (SNPs) and A2756G methionine synthase (MTR) SNP was performed using PCR followed by a high-resolution melting (HRM) analysis. S-adenosyl methionine (SAM) and S-adenosyl homocysteine (SAH) were measured in plasma using high-performance liquid chromatography-tandem mass spectrometry (HPLC/MS/MS). The SNP association analysis was carried out using Fisher's exact test. Statistical analysis was performed using a Mann-Whitney test. *Results*. RNA expression of MTHFR and MTR was significantly higher in patients with PE as compared with controls. Protein, SAM, and SAH levels showed no significant difference between preeclamptic patients and controls. No statistical differences between controls and PE patients were observed with the different SNPs studied. *Conclusion*. The RNA expression of MTHFR and MTR is elevated in placentas of PE patients, highlighting a potential compensation mechanism of the methionine-homocysteine metabolism in the physiopathology of this disease.

## 1. Introduction

Preeclampsia (PE) is a pregnancy-specific disease defined by new-onset hypertension and proteinuria after 20 weeks of gestation. It is present in around 5–10% of all pregnant women worldwide [1] and is associated with an increased risk of maternal and fetal morbidity and mortality, and recently it has been associated with an increased risk of later-life death due to cardiovascular disease for both mother and child [2–4]. 

Several factors have been suggested to be involved in the etiology of PE, but there is consensus that the first step in the pathogenesis of this disease is a defective trophoblastic invasion early in pregnancy [5], which leads to reduced placental perfusion and hypoxia [6]. Nevertheless, it has been proposed that a hypoxic environment is a necessary condition for the invasive phenotype of trophoblast cells [7]. More recently, Lee et al. have described that 2-methoxyestradiol (2-ME), a natural metabolite of 17-*β*-estradiol synthesized by Cathechol-O-Methyltransferase (COMT), induces the differentiation of the endovascular cytotrophoblast cells into its invasive phenotype in the presence of hypoxia [8]. Kanasaki et al. have reported low levels of circulating 2-ME during the third trimester of pregnancy in patients that would later develop PE as compared with controls [9] and our group found the same trend during the early first trimester of pregnancy [10]. With COMT being responsible for methylating 2-Hydroxyestradiol (2-HE) into 2-ME, it has been proposed that the low activity or expression of this enzyme could be involved in the pathogenesis of PE. The normal expression of COMT in placentae of patients with PE [11] and the conflicting results regarding the presence of SNPs that may reduce the activity of this enzyme in these patients [10] force us to search other explanations [12]. The Methionine-homocysteine metabolism (MHM), the process responsible for supplying COMT with the methyl group necessary for 2-ME synthesis, could be an alternative if it is somehow altered, being unable to supply enough methyl groups to sustain adequate concentrations of 2-ME.

It has been shown that alterations in the methionine-homocysteine metabolism (MHM) may be related to the systemic damage that leads to the classical clinical picture of PE [13, 14]. In fact, in normal pregnant women, plasma concentrations of homocysteine are low in the first trimester of pregnancy and reach their lowest level in the second half of gestation [15, 16], whereas hyperhomocysteinemia has been described in women who would develop PE [17–20]. Homocysteine is biosynthesized by the MHM in a process involving the donation of a methyl group by S-adenosylmethionine (SAM), which in turn gets converted into S-adenosylhomocysteine (SAH), a precursor of homocysteine. Through the enzyme methionine synthase (MTR), homocysteine may be remethylated into methionine using 5-methyltetrahydrofolate (5-Methyl-THF) as a cosubstrate. Methylenetetrahydrofolate reductase (MTHFR) is the catalyst for the conversion of 5,10-methylenetetrahydrofolate into 5-methylTHF. Methionine can then be converted into SAM once more to restart the cycle [21]. SAM acts as the principal methyl donor within cells. These methyl groups are key factors in DNA methylation that might control, for example, cellular proliferation, cellular migration, differentiation, cell-to-cell recognition, and other important processes, such as the production of 2-ME through COMT. 

Low levels of SAM may be caused by the presence of single-nucleotide polymorphisms (SNPs) in the genes that code for the enzymes involved in SAM metabolism, such as MTR and MTHFR [22]. Hill et al. recently described that MTHFR modulates the availability of SAM, and the minor “T” allele of the MTHFR C677T SNP has been associated with reduced MTHFR activity, resulting in a decrease in SAM production [23]. Furness et al. suggested that the maternal and placental MTR A2756G allele is an important risk factor for the development of uteroplacental insufficiency [24] though they did not link it to SAM levels and attributed the uteroplacental insufficiency directly to the enzyme. 

We hypothesize that changes in the levels of MTR and MTHFR by the abovementioned SNPs have a negative effect on SAM levels, which, when lowered, contribute to the development of PE. The purpose of this study was to evaluate the bioavailability of SAM as well as of MTR and MTHFR, their respective SNPs, and their relation with preeclampsia. 

## 2. Methods

### 2.1. Study Design

A prospective cohort study was conducted in the Obstetrics and Fetal Medicine Unit from Hospital Parroquial de San Bernardo, Santiago, Chile. Sixteen preeclamptic patients and 32 controls were recruited from this cohort. Both groups consisted of women with singleton gestation and none of them took multivitamins or aspirin during pregnancy. Maternal anthropometric data and blood samples were collected at 11–14-week gestation and at 32–36 weeks. PE was defined as hypertension (arterial pressure (AP) higher or equal to AP 140/90 mmHg on two occasions separated by 6 h or higher or equal to 160/110 mmHg) that occurred after 20 weeks of gestation, in women with previously normal blood pressure, accompanied by proteinuria (300 mg/24 h). Controls, who do not differ in racial origin from PE patients, were healthy subjects without pregnancy complications or chronic medical problems.

Written informed consent was obtained from the women who agreed to participate in the study, which was approved by Hospital Parroquial de San Bernardo and the Universidad de los Andes Ethics Committee.

### 2.2. Tissue and Blood Sample Collection

 Placental tissues were obtained within 15 min of delivery from all of the subjects. Placental samples (*≈*1 cm^3^) were taken from a standardized location, a placental cotyledon between cord insertion and placental border midway, avoiding tissue from areas showing placental infarcts. The tissue was washed in ice-cold physiologic solution (NaCl 0.9%) to remove maternal blood contamination. Samples were processed and stored at −80°C until use for RNA extraction, SNP analysis, and protein quantification. Blood was collected into edetic acid (EDTA) Vacutainer tubes at 11–14 weeks and at 32–34 weeks of gestation, placed in 1.6 mL DNAse/RNAse-free Eppendorf tubes and centrifuged at 1,300 g for 10 min at room temperature. The supernatant was transferred to a fresh 1.6 mL tube and centrifuged once more at 7,200 g for 10 min at room temperature. The supernatant was transferred to a fresh tube and stored at −80°C. 

### 2.3. Protein Quantification

A ~5 mg section from a placental tissue sample (spanning from the maternal to fetal face) was homogenized in a 1.6 mL Eppendorf tube using radioimmunoprecipitation assay (RIPA) lysis buffer (50 mM Tris-HCl pH 8.8; 150 mM NaCl; 0.5% sodium deoxycholate; 0.1% Sodium Dodecyl Sulfate (SDS); 1% Tergitol-type NP-40) with protease inhibitor (cOmplete Mini, Roche). The homogenate was centrifuged at 25,800 g for 30 min at 4°C. The supernatant was carefully transferred to a different 1.6 mL Eppendorf and stored at −20°C. Total protein amount in the supernatant was quantified using BCA Protein Assay Kit (Thermo Scientific) and spectrophotometer NanoDrop 2000 (Thermo Scientific) according to manufacturer's instructions. 20 *μ*g of total protein was loaded for each sample in 12% polyacrylamide gels that were additionally corrected using Coomassie Blue staining and densitometric analysis. An internal control was prepared by pooling 4 different samples (3 controls and 1 PE sample) and was loaded into all gels to allow normalization of data and intergel comparison. 

Protein samples were electrophorised in 12% SDS-polyacrylamide gels and transferred to nitrocellulose membranes (Biorad Laboratories Inc.). Membranes were blocked in 5% (w/v) nonfat dry milk and incubated overnight at 4°C with 1 : 1000 diluted specific monoclonal primary antibodies for MTHFR (Abcam no.ab55530) and MTR (Abcam no.66039). Secondary antibody incubation was performed for 2 hours at room temperature for both genes using a 1 : 1000 diluted peroxidase IgG conjugated anti-mouse secondary antibody (Kirkegaard and Perry Laboratories, Inc.). Specific bands were detected using the ECL Western Blotting Substrates (Thermo Scientific). Blots were scanned and results from densitometric analysis of gels were shown as arbitrary units.

### 2.4. SAM and SAH Quantification

Plasma concentrations of SAM and SAH were measured through high-performance liquid chromatography-tandem mass spectrometry (HPLC-MS/MS) at Innolab Ltda. in Santiago, Chile. Briefly, 500 *μ*L of plasma was vortexed with 312 *μ*L perchloric acid at 100 ml/L and then centrifuged for 10 min at 13,000 rpm. Samples were then transferred to a glass tube, and 140 *μ*L phosphate buffer (pH 11.5) was added along with 1 mL H_2_O. Strata X columns were conditioned with 1 mL methanol and 750 *μ*L lauric acid at 10 mmol/L in 0.1 mol/L NaOH and 1 mL H_2_O. Samples were loaded onto columns and washed with 700 *μ*L H_2_O. The column was eluted with 1 mL ethanol at 0.1% formic acid and the elate was dried under a N_2_ at 40°C. The samples were reconstituted with 200 mL of mobile phase and analyzed in the API 5000 Mass Spectrometer. 

### 2.5. RNA Quantification

Total placental RNA was isolated with MasterPure Complete DNA and RNA Purification Kit (Epicentre Biotechnologies) according to manufacturer's procedures. Total nucleic acid extraction was followed by DNase I treatment to remove DNA contamination. The amount and quality of RNA were assessed using NanoDrop 2000 (Thermo Scientific) at 260/280 with all samples having values between 1.8 and 2.0. Reverse transcriptase PCR was performed on 1 *μ*L of total RNA to obtain complementary DNA using Random Primers (Promega). For gene detection, real-time PCR (qPCR) was performed with the Stratagene Mx3000. For MTHFR: forward primer 5′-ATCACTTGCCCCATCGTC-3′, reverse primer 5′-CATCGTTGTCTTTGATTGGCTC-3′ with probe sequence 5′-ATCACGTCCTTGATCTCCTGTGGC-3′. For MTR: forward primer 5′-ATCTCATCTGGAATAAAGACCCTG-3′, reverse primer 5′-TTCACAAGGGCATACTCAAGG-3′, with probe sequence 5′-CAAGGCACAGGAGGGAAGAAAGTCAT-3′. To quantify the gene expression of each enzyme, a 7-point standard curve (fg/*μ*L), made from a synthetic oligo of the sequence of interest, was run with the samples. Sample concentration was extrapolated from the lineal regression equation of the standard curve used and results are shown in fg/*μ*L ± SEM. 

### 2.6. MTHFR and MTR Genotyping

In order to determine the presence of placental MTHFR and MTR SNPs, tissue DNA was isolated from samples using MasterPure DNA purification kits (Cat no. MC85200, Epicentre Biotechnologies, Madison, USA), following the manufacturer's instructions. After extraction, DNA samples were stored at −20°C. Mutation screening for all three SNPs was performed by qPCR followed by high resolution melting analysis. This technique allows us to differentiate between possible genotypes (both, wild type and mutated homocygote, and heterocygote), through the differences in melting temperature of each haplotype given by the differences in the base pairing (see Figure 1 in supplementary material available online at http://dx.doi.org/10.1155/2013/731962). The primers used for each SNP are MTHFR C677T sense 5′-TGAGGCTGACCTGAAGCACTTGAA-3′, antisense 5′-ATGTCGGTGCATGCCTTCACAAAG-3′; MTHFR A1298C sense 5′-CTTTGGGGAGCTGAAGGACTACTAC-3′, antisense 5′- CACTTTGTGACCATTCCGGTTTG-3′; MTR A2756G sense 5′-GGATGAATACTTTGAGGAAATCATGG-3′, antisense 5′-TGTTTCTACCACTTACCTTGAGAG-3′. Conditions for the PCR reactions were as follows: preincubation at 95°C for 10 min, followed by 45 cycles of denaturation at 95°C for 10 s, 15 s at 62°C annealing, 10 s at 72°C for extension, followed by 60 s denaturation at 95°C, 60 s at 40°C, ending with a 65–95°C at 0.05°C/s high-resolution curve. All samples were run in duplicate using the LightCycler Nano (Roche) and the melting curves were analyzed using the LightCycler Nano Software 1.0 (Roche).

### 2.7. SNP Association Analysis

The SNP association analysis, with PE including the three possible genotypes, was carried out using a Fisher's exact statistic test. The relationship between the SNPs and PE was also tested separately for the subgroups of preeclamptic women against control women, using a dichotomous method (positive-SNP versus negative-SNP). This was done to analyze whether carrying the SNPs was associated with the development of the disease, and a Fisher's exact test was used for this purpose. A threshold of *P* = 0.05 was set for statistical significance of all computed analyses. 

### 2.8. Statistical Analysis

Statistical analysis was performed using a Mann-Whitney test to establish significant statistical differences in parameters between controls and cases concerning mRNA and protein amounts, while Fisher's exact test was carried out to establish significant differences among SNPs. Values are expressed as mean ± SEM. Significant differences were considered when *P* < 0.05. 

## 3. Results

### 3.1. Clinical Characteristics of the Population

 The clinical characteristics of the total group of patients enrolled in this study are shown in Table 1. At 11–14 weeks of gestation, women who developed PE later in gestation showed a significantly higher systolic and diastolic arterial pressure (*P* = 0.003 and *P* = 0.005, resp.) than controls as well as in maternal weight and Body Mass Index (BMI) (*P* = 0.026 and *P* = 0.01, resp.). Gestational age at delivery was also significantly different between controls and preeclampsia patients (*P* < 0.0001). No significant differences were observed in maternal age.

### 3.2. Placental Frequency of MTHFR and MTR Polymorphisms

In order to determine the placental presence of the C677T, A1298C MTHFR, and A2756G MTR gene polymorphisms and their association with PE development,we determined the genotype of placental tissue from control and preeclamptic women.  Table 2 shows the frequencies of the C677T MTHFR, A1298C MTHFR, and A2756G MTR polymorphisms. There was no significant difference between SNP frequencies when comparing controls and PE patients. (MTHFR C677T: control: 72.9% versus PE: 81.3%; *P* = 0.725; MTHFR A1268C: control: 87.5% versus PE: 62.5%; *P* = 0.064; MTR A2756G: control: 50.0% versus PE: 37.5; *P* = 0.542). 

### 3.3. MTHFR, MTR RNA Levels

**Figure 1:**
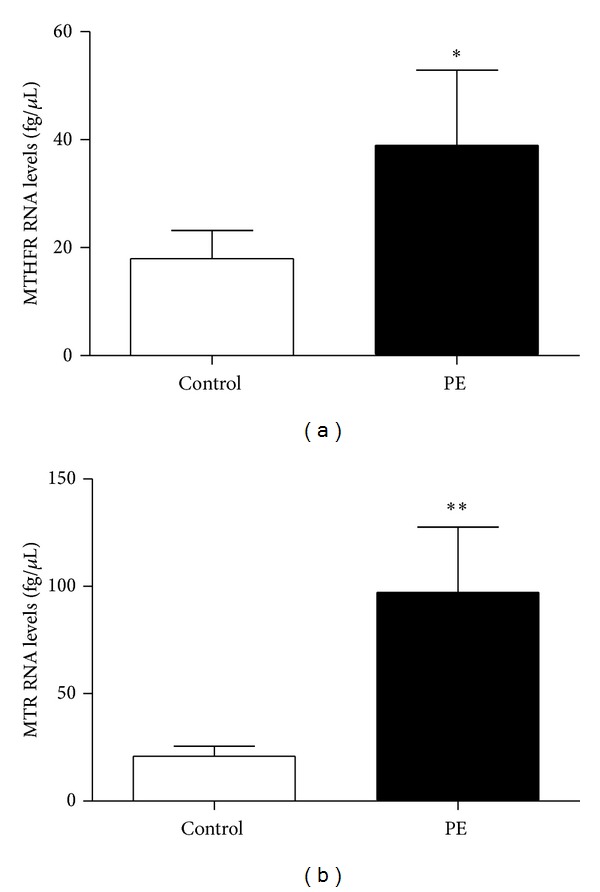
Elevated levels of mRNA for (a) MTHFR and (b) MTR in women who developed PE. Levels were measured by RT-PCR followed by qPCR. Values are ± SEM from 32 control and 16 PE patients: *P < 0.05, **P < 0.01.

MTHFR and MTR RNA expression was measured in term placentas from all patients. We used RT-PCR followed by qPCR to calculate the relative concentrations of the different RNA. As shown in Figure 1, women who developed PE had significantly higher RNA expression for MTHFR and MTR (MTHFR: 18.17 ± 5.26 (SEM) versus 38.99 ± 13.89 fg/*μ*L, *P* = 0.02; MTR: 21.00 ± 4.79 versus 97.13 ± 30.40 fg/*μ*L, *P* = 0.0002).

### 3.4. MTHFR and MTR Protein Levels

**Figure 2:**
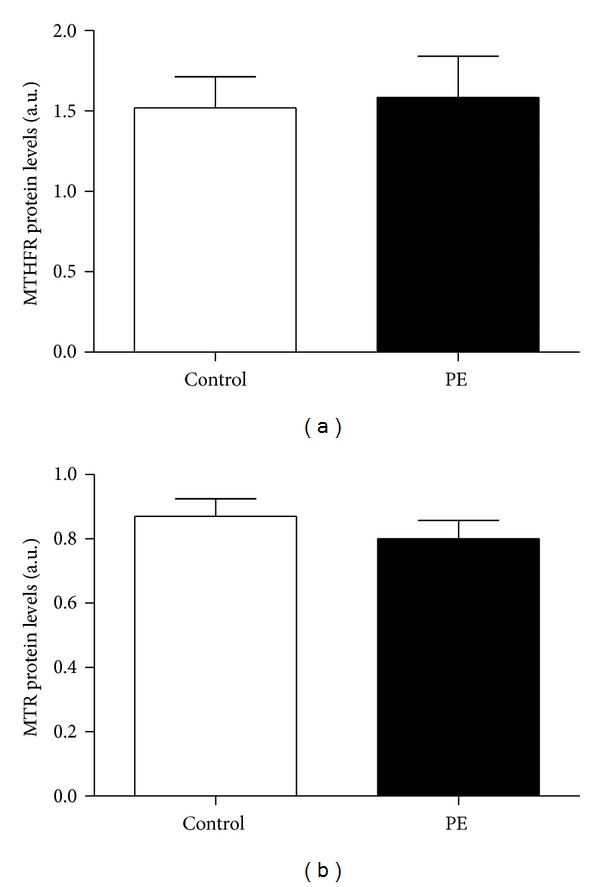
(a) MTHFR and (b) MTR protein levels analyzed from placental tissue by western blot. Values are ± SEM from 32 controls and 16 PE patients.

Protein content was measured in placental tissue from all patients in this study to determine if there was any association between altered protein content and the development of PE.  Figure 2 shows the relative protein content for MTHFR and MTR with no significant differences between controls and PE patients (MTHFR: 1.52 ± 0.19 (SEM) versus 1.58 ± 0.26 arbitrary units (a.u.), *P* = 0.44; MTR: 0.87 ± 0.05 versus 0.80 ± 0.05 a.u., *P* = 0.55). 

### 3.5. SAM and SAH Levels

**Figure 3:**
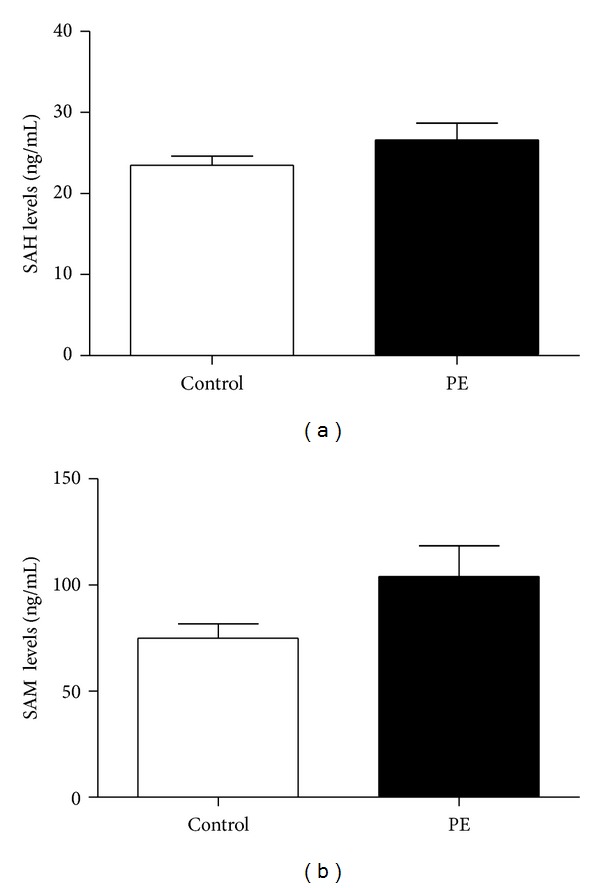
(a) SAM and (b) SAH levels obtained form maternal serum 4–6 weeks prior to delivery. Amounts measured by HPLC-MS/MS. Values are ± SEM from 32 controls and 16 PE patients.

Metabolite concentrations were measured in maternal serum samples obtained 4–6 weeks before delivery from all patients in the study to establish the relationship between SAH produced and SAM available in PE patients and control.  Figure 3 shows the amount of SAM and SAH with no significant differences between control and PE patients (SAM: 75.36 ± 6.69 (SEM) versus 104.1 ± 14.34 ng/mL, *P* = 0.08; SAH: 23.58 ± 1.15 versus 26.60 ± 2.07 ng/mL, *P* = 0.32; SAH/SAM: 0.43 ± 0.06 versus 0.34 ± 0.06, *P* = 0.16).

## 4. Discussion

In this study we tested if the development of PE was connected to an alteration in the enzymes involved in the MHM. As early as 11–14 weeks of gestation, the PE cohort displayed significantly higher systolic and diastolic blood pressure (*P* > 0.05) as well as significantly higher maternal weight and BMI (*P* > 0.05) (Table 1), and all displayed clinical features that have been described previously as predictors of PE or related to the development of this disease [25–27]. The case and control cohorts used in this study are, thus, well characterized and defined with difference between them not only present in the third trimester, but also in the first trimester of pregnancy. 

Recently, we reported that low 2-ME plasma concentrations at 11–14 weeks of gestation are associated with the development of preeclampsia, but these low concentrations cannot be explained by abnormal expression of COMT [11], and there are conflicting results regarding the presence of SNPs that could reduce the activity of COMT in patients with PE. In this study, we tested the hypothesis that these low concentrations were associated with an alteration in the MHM key enzymes and/or SAM and SAH plasma concentrations and PE development. 

First, we evaluated if there was an association between the presence of placental MTHFR and MTR functional polymorphisms and the development of PE. Analysis through PCR followed by high-resolution melting revealed no significant difference in the frequency of SNPs between PE patients and the control group. Robertson et al., however, conducted a systematic review of 25 studies (*n* = 11, 183) to investigate the association between inherited thrombophilias and the risk of adverse pregnancy outcomes, including PE. They reported that the risk of PE was significantly higher in the presence of MTHFR homozygosity (MTHFR T/T); the MTHFR C677T SNP had an odds ratio of 1.37 (95% CI, 1.07–1.76) for developing PE [28]. The reduced number of patients in our cohort could explain this difference, but until a larger study that tests the penetration of these SNPs in our normal population is carried out, we will be unable to give a definitive answer. 

Second, we evaluated if there was a difference in RNA and protein content for these key enzymes of the MHM that may be associated with the development of PE. While RNA expression of MTHFR and MTR was significantly higher in placental tissue obtained from PE patients, there was no difference in protein content for these two enzymes between the studied groups. This may be explained by the time at which the samples were collected; RNA and protein having differences in synthesis time. While RNA is increased, the protein content could remain low, and when RNA is translated into protein, RNA may decrease while protein content gradually increases. Thus, though the RNA expression proved to be significantly different, the change may have not been yet reflected in protein content or a regulating step was present not during transcription, but during translation of the RNA into protein [29]. Also, it has been shown that small-interference RNA (siRNA) and/or micro-RNA (miRNA) are other possible mechanisms to regulate translation process [30]. More studies are needed to elucidate if altered siRNA or miRNA expressions are involved in differential RNA-protein levels observed between PE and control patients. Nevertheless, the high expression of RNA of these enzymes in patients with PE reported here could be reflecting a compensatory mechanism that, for example, ensures normal MHM enzyme levels to maintain the role of the MHM cycle in pregnancy physiology. 

Finally, we measured the plasma concentrations of SAM and SAH in order to determine if there was an alteration in these key metabolites in PE patients compared with controls. With the observed higher mRNA expression, we expected decreased concentrations of SAM and increased SAH and 2-ME levels. No differences, however, were identified in the concentrations of SAM or SAH between PE patients and the control groups. One possible explanation for this result is the origin of our cohort. The Chilean population has folic acid supplemented in the diet, and most pregnant women take additional folic acid supplementation (our patients were not the exception), thus, providing adequate amounts of methionine for conversion into SAM. The measurement of plasma folic acid concentrations may have provided further support to this explanation. The observation that SAM concentrations are not reduced in patients that develop PE, however, suggests that other processes contribute to reducing 2-ME. 

The data obtained in this study indicate that MHM is not altered in women who subsequently develop PE. Nevertheless, 2-ME concentrations are decreased as early as 11–14 weeks of gestation [10]. When interpreting the data, however, it must be taken into account that protein and RNA content, as well as the presence of SNPs, were analyzed in placenta while the SAM and SAH were measured in maternal plasma. It is possible that the maternal aspect of the cycle is sustaining adequate concentrations of SAM and SAH. Nevertheless, it is likely that another pathway(s) account for the lower levels of 2-ME in preeclamptic patients. 2-ME is a natural metabolite of 17-*β*-estradiol; therefore, reduced substrate availability and/or conversion to 2ME may contribute to reduced biosynthesis. The aromatase pathway is responsible for the conversion of androgens into estrogens; more specifically, testosterone and androstenedione into 17-*β*-estradiol and estrone, respectively [31]. 17-*β*-estradiol is hydroxylated at the 2-position by the cytochrome p450, resulting in the synthesis of 2-HE, that subsequently undergoes O-methylation of the catechol ring by COMT to ultimately result in 2-ME. It has also been suggested that aromatase is the rate-limiting enzyme in the production of 2-ME [32], making it a potential key enzyme to study in the development of PE as alterations in its bioavailability may affect the levels of 2-ME. Further studies will be needed to test this hypothesis.

## 5. Conclusion

In conclusion, this study shows that women who develop PE are clinically distinguishable at 11–14 weeks of gestation. Key enzyme RNA expression is increased in preeclamptic patients, but this change is not reflected at the protein content. SNP frequencies as well as SAM and SAH concentrations display no difference between women with PE and controls. These results highlight a potential role of the MHM as a compensation mechanism in the presence of low levels of 2-ME. 

## Supplementary Material

Representative images of the MTHFR C677T SNP of the difference plot and normalized plot obtained through qPCR-High-Resolution melting analysis. Every base change affects the melting temperature of the double stranded DNA. These slight changes can be detected through High-Resolution melting, giving a slightly different melting curve for each base sequence, allowing for detection of specific SNPs.

## Figures and Tables

**Table 1:** Clinical characteristics of controls and preeclampsia patients, at 11–14 weeks of gestation.

	Control patients (*n* = 32)	Preeclampsia patients (*n* = 16)	*P* value
Maternal age (years)	25.5 ± 1.2	29.9 ± 1.9	0.0835
Gestation age at delivery (weeks)	39.3 ± 0.2	36.6 ± 1.0	<0.0001***
At 11–14 weeks			
Systolic pressure (mmHg)	107.3 ± 1.9	121.4 ± 3.6	0.0029**
Diastolic pressure (mmHg)	64.7 ± 1.4	72.3 ± 3.8	0.0053*
Maternal weight (kg)	64.3 ± 2.2	75.1 ± 4.4	0.0263*
Body mass index (kg/m^2^)	25.4 ± 0.9	30.4 ± 1.7	0.0099*

Values are given as mean ± SEM. Statistical significance was assessed using Mann Whitney test. *P < 0.05; **P < 0.005; ***P < 0.001.

**Table 2:** Frequencies of genotypes of the methylenetetrahydrofolate reductase (MTHFR) and methionine synthase (MTR) gene in controls and preeclamptic patients.

Genotypes	Control patients (*n* = 32)	Preeclampsia patients (*n* = 16)	*P* value	OR	(95% Conf. interval)
*MTHFR C677T *					
Wild Type	9 (28.1%)	3 (18.8%)	0.725	1.69	[0.389–7.400]
Heterozygote/homozygote	23 (71.9%)	13 (81.2%)			
*MTHFR A1268C *					
Wild Type	4 (12.5%)	6 (37.5%)	0.063	0.238	[0.055–1.022]
Heterozygote/homozygote	28 (87.5%)	10 (62.5%)			
*MTR A2756G *					
Wild Type	16 (50.0%)	10 (62.5%)	0.542	0.6	[0.176–2.046]
Heterozygote/homozygote	16 (50.0%)	6 (37.5%)			

MTHFR C677T: wild type (C/C), heterozygote (C/T) and homozygote (T/T); MTHFR A1268C: wild type (A/A), heterozygote (A/C), homozygote (C/C); MTR A2756G: wild type (A/A), heterozygote (A/G), homozygote (G/G). Data show the number of control and preeclamptic patients with or without the polymorphism. Statistical significance was assessed using Fisher's exact test.

## References

[1] Duley L (2009). The Global Impact of Pre-eclampsia and Eclampsia. *Seminars in Perinatology*.

[2] Bellamy L, Casas J-P, Hingorani AD, Williams DJ (2007). Pre-eclampsia and risk of cardiovascular disease and cancer in later life: systematic review and meta-analysis. *British Medical Journal*.

[3] McDonald SD, Malinowski A, Zhou Q, Yusuf S, Devereaux PJ (2008). Cardiovascular sequelae of preeclampsia/eclampsia: a systematic review and meta-analyses. *American Heart Journal*.

[4] Mongraw-Chaffin ML, Cirillo PM, Cohn BA (2010). Preeclampsia and cardiovascular disease death: prospective evidence from the child health and development studies cohort. *Hypertension*.

[5] Rauramo I, Forss M (1988). Effect of exercise on placental blood flow in pregnancies complicated by hypertension, diabetes or intrahepatic cholestasis. *Acta Obstetricia et Gynecologica Scandinavica*.

[6] Pijnenborg R, Vercruysse L, Brosens I (2011). Deep placentation. *Best Practice and Research*.

[7] Rodesch F, Simon P, Donner C, Jauniaux E (1992). Oxygen measurements in endometrial and trophoblastic tissues during early pregnancy. *Obstetrics and Gynecology*.

[8] Lee SB, Wong AP, Kanasaki K (2010). Preeclampsia: 2-Methoxyestradiol induces cytotrophoblast invasion and vascular development specifically under hypoxic conditions. *American Journal of Pathology*.

[9] Kanasaki K, Palmsten K, Sugimoto H (2008). Deficiency in catechol-O-methyltransferase and 2-methoxyoestradiol is associated with pre-eclampsia. *Nature*.

[10] Pérez-Sepúlveda A, Torres MJ, Valenzuela FJ, Larraín R, Figueroa-Diesel H (2012). Low 2-methoxyestradiol levels at the first trimester of pregnancy are associated with the development of pre-eclampsia. *Prenat Diagn*.

[11] Palmer K, Saglam B, Whitehead C, Stock O, Lappas M, Tong S (2011). Severe early-onset preeclampsia is not associated with a change in placental catechol O-methyltransferase (COMT) expression. *American Journal of Pathology*.

[12] Perez-Sepulveda A, Espana-Perrot PP, Norwitz ER, Illanes SE Metabolic pathways involved in 2-methoxyestradiol synthesis and their role in preeclampsia.

[13] López-Quesada E, Vilaseca MA, Lailla JM (2003). Plasma total homocysteine in uncomplicated pregnancy and in preeclampsia. *European Journal of Obstetrics Gynecology and Reproductive Biology*.

[14] Shenoy V, Kanasaki K, Kalluri R (2010). Pre-eclampsia: connecting angiogenic and metabolic pathways. *Trends in Endocrinology and Metabolism*.

[15] Cikot RJLM, Steegers-Theunissen RPM, Thomas CMG, De Boo TM, Merkus HMWM, Steegers EAP (2001). Longitudinal vitamin and homocysteine levels in normal pregnancy. *British Journal of Nutrition*.

[16] Murphy MM, Scott JM, McPartlin JM, Fernandez-Ballart JD (2002). The pregnancy-related decrease in fasting plasma homocysteine is not explained by folic acid supplementation, hemodilution, or a decrease in albumin in a longitudinal study1-3. *American Journal of Clinical Nutrition*.

[17] Powers RW, Evans RW, Majors AK (1998). Plasma homocysteine concentration is increased in preeclampsia and is associated with evidence of endothelial activation. *American Journal of Obstetrics and Gynecology*.

[18] Wang J, Trudinger BJ, Duarte N, Wilcken DE, Wang XL (2000). Elevated circulating homocyst(e)ine levels in placental vascular disease and associated pre-eclampsia. *British Journal of Obstetrics and Gynaecology*.

[19] D’Anna R, Baviera G, Corrado F, Ientile R, Granese D, Stella NC (2004). Plasma homocysteine in early and late pregnancies complicated with preeclampsia and isolated intrauterine growth restriction. *Acta Obstetricia et Gynecologica Scandinavica*.

[20] Makedos G, Papanicolaou A, Hitoglou A (2007). Homocysteine, folic acid and B12 serum levels in pregnancy complicated with preeclampsia. *Archives of Gynecology and Obstetrics*.

[21] Födinger M, Hörl WH, Sunder-Plassmann G (2000). Molecular biology of 5,10-methylenetetrahydrofolate reductase. *Journal of Nephrology*.

[22] Sharma P, Senthilkumar RD, Brahmachari V (2006). Mining literature for a comprehensive pathway analysis: a case study for retrieval of homocysteine related genes for genetic and epigenetic studies. *Lipids in Health and Disease*.

[23] Hill LD, York TP, Kusanovic JP (2011). Epistasis between COMT and MTHFR in maternal-fetal dyads increases risk for preeclampsia. *PLoS ONE*.

[24] Furness DLF, Fenech MF, Khong YT, Romero R, Dekker GA (2008). One-carbon metabolism enzyme polymorphisms and uteroplacental insufficiency. *American Journal of Obstetrics and Gynecology*.

[25] Poon LCY, Kametas NA, Valencia C, Chelemen T, Nicolaides KH (2011). Hypertensive disorders in pregnancy: screening by systolic diastolic and mean arterial pressure at 11–13 weeks. *Hypertension in Pregnancy*.

[26] Sohlberg S, Stephansson O, Cnattingius S, Wikström A-K (2012). Maternal body mass index, height, and risks of preeclampsia. *American Journal of Hypertension*.

[27] Syngelaki A, Bredaki FE, Vaikousi E, Maiz N, Nicolaides KH (2011). Body mass index at 11-13 weeks’ gestation and pregnancy complications. *Fetal Diagnosis and Therapy*.

[28] Robertson L, Wu O, Langhorne P (2006). Thrombophilia in pregnancy: a systematic review. *British Journal of Haematology*.

[29] Vogel C, Marcotte EM (2012). Insights into the regulation of protein abundance from proteomic and transcriptomic analyses. *Nature Reviews Genetics*.

[30] Zhang B, Wang Q, Pan X (2007). MicroRNAs and their regulatory roles in animals and plants. *Journal of Cellular Physiology*.

[31] Czajka-Oraniec I, Simpson ER (2010). Aromatase research and its clinical significance. *Endokrynologia Polska*.

[32] Hertig A, Liere P, Chabbert-Buffet N (2010). Steroid profiling in preeclamptic women: evidence for aromatase deficiency. *American Journal of Obstetrics and Gynecology*.

