# Supplementary figures and images for: Levels of Key Enzymes of Methionine-Homocysteine Metabolism in Preeclampsia
Source: Biomed Res Int. 2013 Aug 20;2013:731962. doi: 10.1155/2013/731962 (PMC3762171; doi:10.1155/2013/731962)

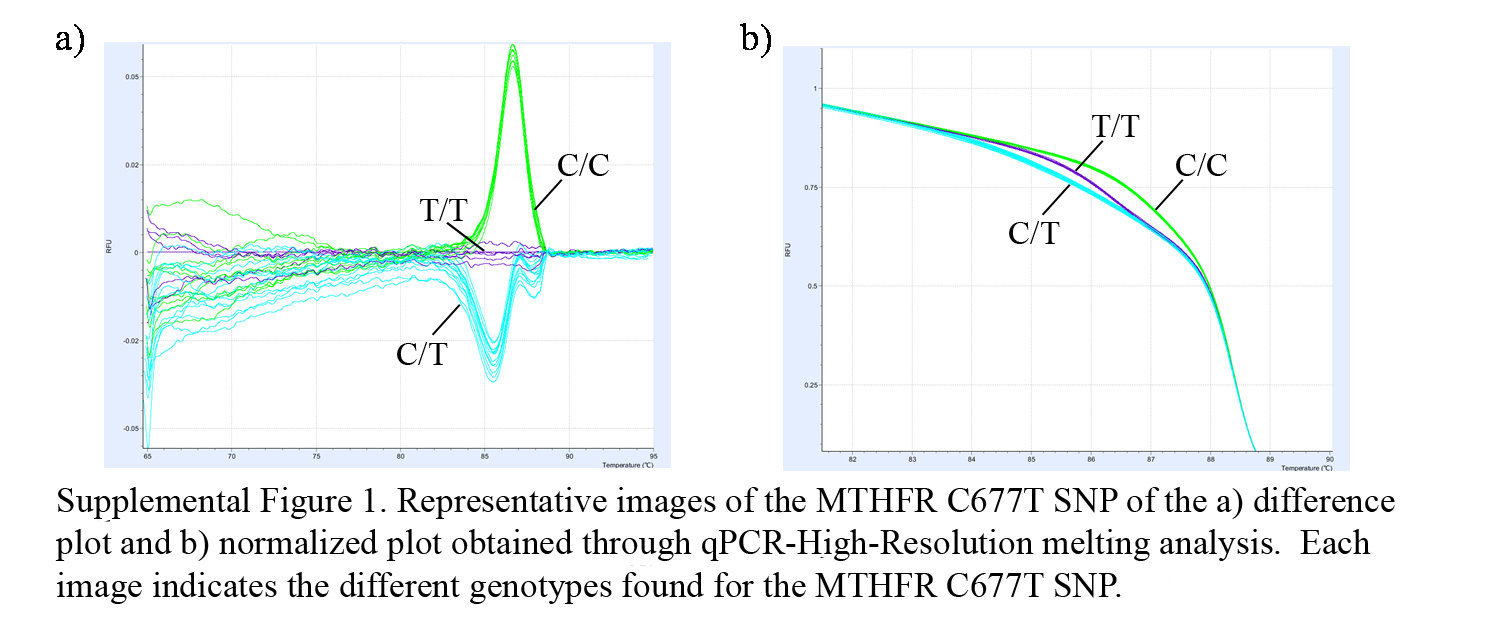

Supplement: Supplementary file 1 — Representative images of the MTHFR C677T SNP of the difference plot and normalized plot obtained through qPCR-High-Resolution melting analysis. Every base change affects the melting temperature of the double stranded DNA. These slight changes can be detected through High-Resolution melting, giving a slightly different melting curve for each base sequence, allowing for detection of specific SNPs. [file 731962.f1.jpg]
